# Circulating proteomic signature for detection of biomarkers in bladder cancer patients

**DOI:** 10.1038/s41598-020-67929-z

**Published:** 2020-07-03

**Authors:** Taoufik Nedjadi, Hicham Benabdelkamal, Nada Albarakati, Afshan Masood, Ahmed Al-Sayyad, Assim A. Alfadda, Ibrahim O. Alanazi, Adel Al-Ammari, Jaudah Al-Maghrabi

**Affiliations:** 1 King Abdullah International Medical Research Center, King Saud Bin Abdulaziz University for Health Sciences, PO Box 9515, Jeddah, 21423 Saudi Arabia; 2 Proteomics Resource Unit, Obesity Research Center, College of Medicine, King Saud University, Riyadh, Saudi Arabia; 3 Department of Urology, King Abdulaziz University, Jeddah, Saudi Arabia; 4 National Center for Biotechnology (NCBT), Life Science and Environment Research Institute, King Abdulaziz City for Science and Technology (KACST), Riyadh, Saudi Arabia; 5 Department of Urology, King Faisal Specialist Hospital and Research Center, Jeddah, Saudi Arabia; 6 Department of Pathology, King Abdulaziz University, Jeddah, Saudi Arabia

**Keywords:** Bladder cancer, Tumour biomarkers

## Abstract

The identification of clinically-relevant early diagnostic and prognostic protein biomarkers is essential to maximize therapeutic efficacy and prevent cancer progression. The aim of the current study is to determine whether aberrant plasma protein profile can be applied as a surrogate tool for early diagnosis of bladder carcinoma. Plasma samples from patients with low grade non-muscle invasive bladder cancer and healthy controls were analyzed using combined 2D-DIGE and mass-spectrometry to identify differentially expressed proteins. Validation was performed using western blotting analysis in an independent cohort of cancer patients and controls. Fifteen differentially-expressed proteins were identified of which 12 were significantly up-regulated and three were significantly down-regulated in tumors compared to controls. The Ingenuity Pathways Analysis revealed functional connection between the differentially-expressed proteins and immunological disease, inflammatory disease and cancer mediated through chemokine and cytokine signaling pathway and NF-kB transcription factor. Among the three validated proteins, haptoglobin was able to distinguish between patients with low grade bladder cancer and the controls with high sensitivity and specificity (AUC > 0.87). In conclusion, several biomarker proteins were identified in bladder cancer. Haptoglobin is a potential candidate that merit further investigation to validate its usefulness and functional significance as potential biomarkers for early detection of bladder cancer.

## Introduction

Bladder carcinoma (BC) is the second most common cancer of the genitourinary tract and a leading cause of mortality worldwide. According to a recent data, an estimated 450,000 persons developed BC and more than 199,000 persons died of BC in the year 2018^1^. Early diagnosis of bladder cancer has a better prognosis with a 10-year disease-free survival of 86% for non-muscle invasive bladder cancer (NMIBC) (stage T0). However, due to the absence of reliable prognostic markers, recurrence rates fluctuate between 30 and 70% with as high as 10–30% rates of progression to muscle-invasive bladder cancer (MIBC) phenotype for high-risk patients^2,3^. This situation necessitates vigilant management protocol through frequent cystoscopy/cytology examinations and different treatment modalities. The extended treatment and long-term follow-up make bladder cancer treatment one of the most expensive per patient among all cancers^4^. Therefore, biomarkers for early stage screening of non-muscle invasive bladder cancer is desperately needed as it will help in early diagnosis, preventing disease progression to a life-threatening phenotype (MIBC) and will eventually lower the associated costs of treatment. Furthermore, early diagnosis of bladder cancer would be of great importance in discerning patients with high grade cancer from those with low grade cancer and in selecting the appropriate therapeutic approaches.

Despite development and FDA-approval of several urine and blood-based biomarker kits for appraisal of bladder cancer, cystoscopy and urine cytology remain the gold standard clinical approaches for bladder cancer detection and surveillance^5,6^. Both methods have several pitfalls of being invasive, expensive, with inadequate sensitivity and specificity especially for low grade tumors and carcinoma in situ lesions^7^. Moreover none of the biomarker kits have demonstrated sufficient sensitivity/specificity to replace the gold standard methods in bladder cancer^8,9^.

It is well perceived that proteins are the key regulators that execute cellular functions. Therefore the proteomic signatures will most closely reflect the physiological and the pathological alterations that occur in the carcinogenic process^10,11^. Blood derivatives, either serum or plasma, have been considered as promising sources for developing proteomic protocols and the discovery of non-invasive biomarkers as blood samples are relatively easy to obtain and are rich with circulating aberrant proteins that can be used as tumor biomarkers^12^. Two dimensional gel-electrophoresis (2D-E) combined with mass spectrometry (MS) have evolved greatly and are capable of analyzing and identifying multiple differentially expressed proteins with high sensitivity and specificity^13,14^. Recent years have witnessed tremendous expansion in circulating biomarkers analysis by liquid chromatography/MS to develop bladder cancer biomarkers for diagnosis and/ or prognosis^15–17^. In this context several single biomarker molecules, with mechanistic relevance to bladder cancer, have been identified including complement C3b, CYFRA 21-1, ApoA4, ApoE and soluble E-cadherin^17–19^. Also, extensive research has been performed on multi-markers model which has led to the identification of panels of biomarkers that are differentially regulated in bladder carcinoma^20,21^. This approach may provide a comprehensive understanding of the complex mechanisms underlying bladder tumorogenesis. However there is still relative lack of comprehensive studies exploring the landscape of differentially-expressed proteins in bladder cancer. In this study, we used 2D-DIGE followed by MALDI-TOF mass-spectrometry to identify potential plasma biomarkers and explore proteome profile changes that differentiate between a cohort of patients with low grade non-muscle invasive bladder cancer and healthy controls which may help in the clinical setting and the management of BC.

## Results

### Samples

Plasma samples (discovery set and validation set) were grouped into two groups: the first one for patient with low grade non-muscle invasive tumors and the second group comprise healthy controls (Table 1). All plasma samples were depleted to reduce the complexity and enrich the proteins that might be used as biomarkers (Supplementary Fig. 1). For such reason, plasma pre-fractionation strategy was undertaken using immuno-depletion kit that removes 20 of the most abundant proteins, including immunoglobulins and albumin, from the plasma and the final fractions were collected. This step removes more than 95% of the high-abundance proteins in serum; hence increase the number of detectable spots. After depletion protein fractions from each of the tumor group and the healthy group were labeled with Cy3 and Cy5 Dyes respectively. In order to remove gel-to-gel variation in 2D-DIGE, control sample (internal standard) was labeled with Cy2 dye and run in parallel with the other gels.

**Table 1:** Characteristics of low-grade cancer vs. healthy controls.

Characteristics	Cancer patients	Healthy controls
**Number of patients**		
Discovery	4	4
Validation	10	18
Age (mean/range)	62/(50–73)	51/(43–60)
Weight (mean/range)	68/(55–80)	74/(59–88)
**Gender**		
Male	80%	87%
Female	20%	13%
Cancer grade low	100%	N/A
**Stage**		
pTa	33.3%	N/A
pT1	67.7%	N/A

### Identification of candidate biomarkers

Comparisons of individual plasma samples (discovery set) from patients with low grade BC (n = 4 patients) and healthy controls (n = 4) were equally loaded, labeled and separated on 2D-DIGE. Differentially expressed protein spots were excised and identified by MALDI-TOF mass spectrometry. Analysis of the discovery cohort revealed a total of 980 spots consistently detected and mapped on all gels between the two groups by 2D-DIGE. Representative 2D-DIGE gels of each condition are shown in Fig. 2A–D. A total of 22 protein spots with a statistical significant change (ANOVA-test p < 0.05; fold-change ≥ 1.5) in abundance between the patients with low grade BC and control groups were identified. These spots were selected and further analyzed by MALDI TOF. 15 protein spots were successfully identified and matched using the MASCOT PMF to entries in the SWISS-PROT database with high confidence. Of the 15 spots identified, 12 spots were up and three down in patients with low grade bladder cancer in comparison to controls (Table 2).

**Figure 1:**
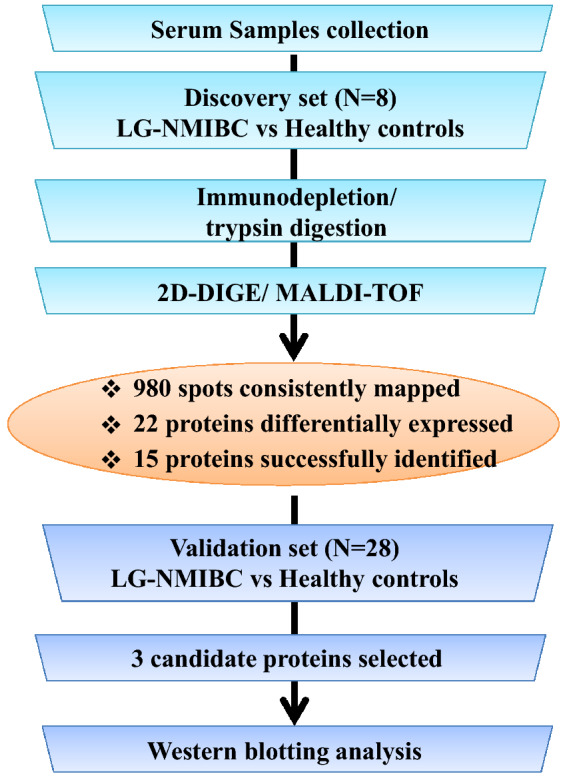
Schematic representation of the workflow of plasma proteomic analysis. The study schema is illustrated with the patient populations and methods used. Plasma protein biomarker discovery (N = 8) was guided by plasma‐based proteomics followed by 2D-DIGE mass spectrometry. Selected protein candidates were validated in an independent cohort (N = 28) by western blotting analysis.

**Figure 2:**
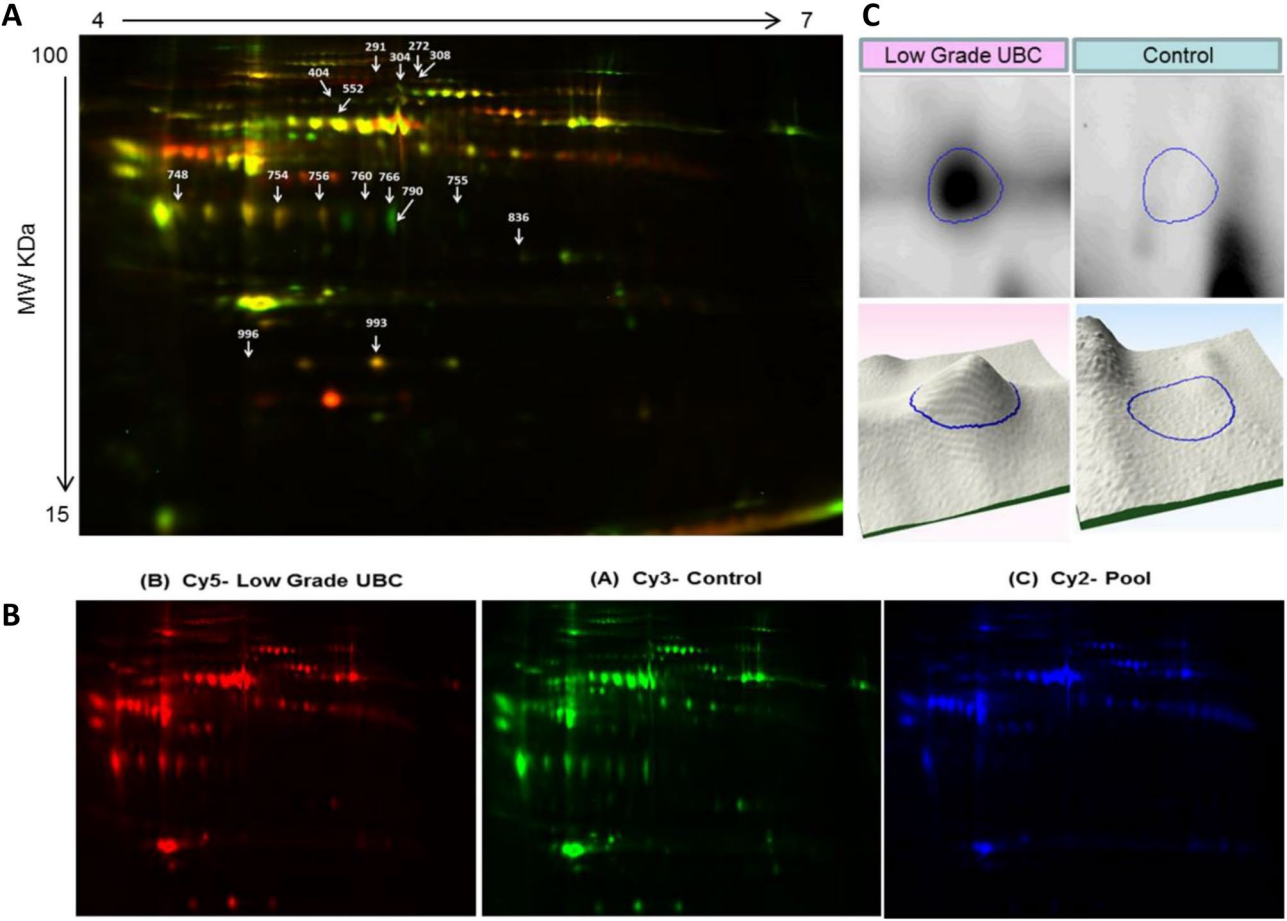
The proteome maps obtained from two-dimensional proteome map analysis of plasma samples from the discovery bladder cancer patients/ healthy control cohorts. (**A**) A representative overlap 2D-DIGE image. The differentially expressed spots are denoted by arrows. (**B**) Images for low grade tumor, healthy control and internal standard were labeled with Cy3, Cy5 and Cy2 dyes respectively. (**C**) Panel depicted an example of protein spot 755 (Integrator complex subunit 10) abundance by 2D and 3D overexpressed in tumor compared to control.

**Table 2:** List of identified differentially expressed proteins in low grade bladder cancer serum samples identified using 2D DIGE and MALDI-TOF-MS analysis.

	Spot no.	Accession no.	Protein name	Pi	Cov%	MW	p-value (ANOVA)	Fold change	UP/down regulated
1	552	P02790	Hemopexin	6.55	33	52,385	0.038	1.7	UP
2	993	P00738	Haptoglobin	6.3	41	45,861	0.038	4.8	UP
3	748	Q8IXV7	Kelchdomain-containing protein 8B	8.61	28	38,165	0.039	3.6	UP
4	754	P00738	Haptoglobin	6.3	19	65,861	0.038	4.3	UP
5	836	Q9ULT8E3 ubiquitin-protein ligase HECTD1	5.29	8	292,379	0.044	1.7	UP	
6	766	Q9Y6K1DNA (cytosine-5)-methyltransferase3A	619	17	103,390	0.047	2.8	UP	
7	756	P00738	Haptoglobin	6.3	28	4,586	0.050	3.5	UP
8	404	P00736	Complement C1r subcomponent	5.82	20	81,606	0.011	2.3	UP
9	291	P00450	Ceruloplasmin	5.44	43	122,983	0.008	2.3	Down
10	996	Q9Y5U9	Immediate early response 3-interacting protein1	7.96	19	9,020	0.012	3.9	UP
11	304	P01024	Complement C3	6.39	24	88,569	0.004	2.1	Down
12	755	Q9NVR2	Integrator complex subunit 10	724	25	83,209	0.005	3	UP
13	790	P00738	Haptoglobin	6.3	31	4,586	0.043	3.6	UP
14	308	P13671	Complement component C6	6.39	27	108,367	0.001	2.3	Down
15	760	P00738	Haptoglobin	6.3	33	45,861	0.057	3.7	UP

### Validation of differentially expressed plasma proteins

**Figure 3:**
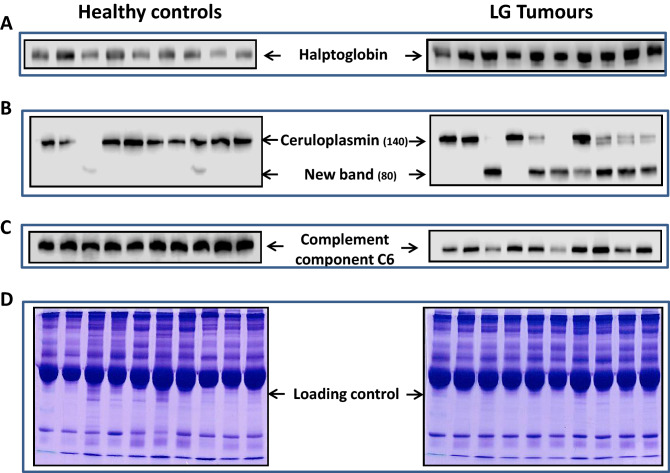
Validation of selected differentially expressed proteins using independent validation cohorts. Immunoblot analysis using of complement component C6 proteins (**A**), ceruplasmin (**B**) and haptoglobin (**C**). Equal protein loading was confirmed with Coomassie staining of plasma samples. Densitometry analysis of each protein normalized to β-actin represents the relative protein expression values. Error bars represent standard error of the mean. *p 0.05, **p 0.01, unpaired t-test. n = three replicates of three independent experiments for each group.

Although several differentially-expressed proteins were identified only three proteins selected for further validation for many reasons. Most importantly, the selected proteins exhibited significant p-value of increase or decrease in cancer compared to healthy controls, because of the higher protein score and sequence coverage of ceruloplasmin, haptoglobin and C6 compared with the other proteins identified and also due to time restraint and shortage of technical staff. Furthermore, published data indicated that changes in serum levels of all three proteins are remarkably altered in solid tumours and are associated with poor prognosis. Western blotting analysis was carried out to validate the data using plasma samples from independent cohorts (validation set) of low grade bladder tumors (n = 18) and healthy controls (n = 10). We focused on three proteins: two down-regulated proteins (ceruloplasmin and C6) and one up-regulated protein (haptoglobin) because these proteins show significant p-value difference between cancer and healthy controls. The experiments were repeated three times for each target protein and the results gathered for all three proteins confirm the trend seen in 2D-DIGE analysis. Haptoglobin level was significantly increased (*p* = 0.0006) in low grade bladder cancer patients when compared to healthy control group (Fig. 3A). This is consistent with 2D-DIGE data (Table 2). Western blots of plasma samples probed with the ceruloplasmin antibody exhibited the tendency observed in 2D-DIGE and showed reduced expression of the 140 kDa isoform in low grade bladder cancer patients when compared to healthy controls (Fig. 3B). Interestingly, extra fragment (80 kDa) was observed across all tumor patients with low 140 kDa ceruloplasmin isoform expression. The intensity of the band (80 kDa) was inversely proportional to the level of the140 kDa band. The 80 kDa isoform is weakly expressed in only two controls but completely absent in the rest of healthy controls (Supplementary Fig. 2). This suggests that the uncharacterized ceruloplasmin isoform (80 kDa) may arise from a pre-spliced transcript or through proteolytic cleavage of the original protein. Validation for complement C6 expression in an independent cohort (Fig. 3C) demonstrated significant reduction in C6 protein levels in low grade bladder cancer patients when compared to healthy controls (*p* = 0.046) which is consistent with the 2D-DIGE protein expression data. Equal protein loading was confirmed using Coomassie blue staining (Fig. 3D). Immunoblot band intensity for haptoglobin, ceruloplasmin and complement protein C6 was measured by densitometry using ImageJ software and presented as relative band intensity (Supplementary Fig. 3).

### Principal component analyses (PCA) analyses

We then sought to investigate the total protein abundance variation between both cohorts. All gels images were grouped into two groups according to whether they have cancer or not and principal component analysis was performed using Progenesis SameSpots software. Statistically significant protein spots that were identified by MS were selected for such analysis. PCA plot of the two first principal components revealed that the two groups (cancer and controls) clustered distinctly from one another and the selected spots exhibit 52% abundance variability between the two groups (Supplementary Fig. 4A,B).

### Interaction and functional characteristics of key proteins

**Figure 4:**
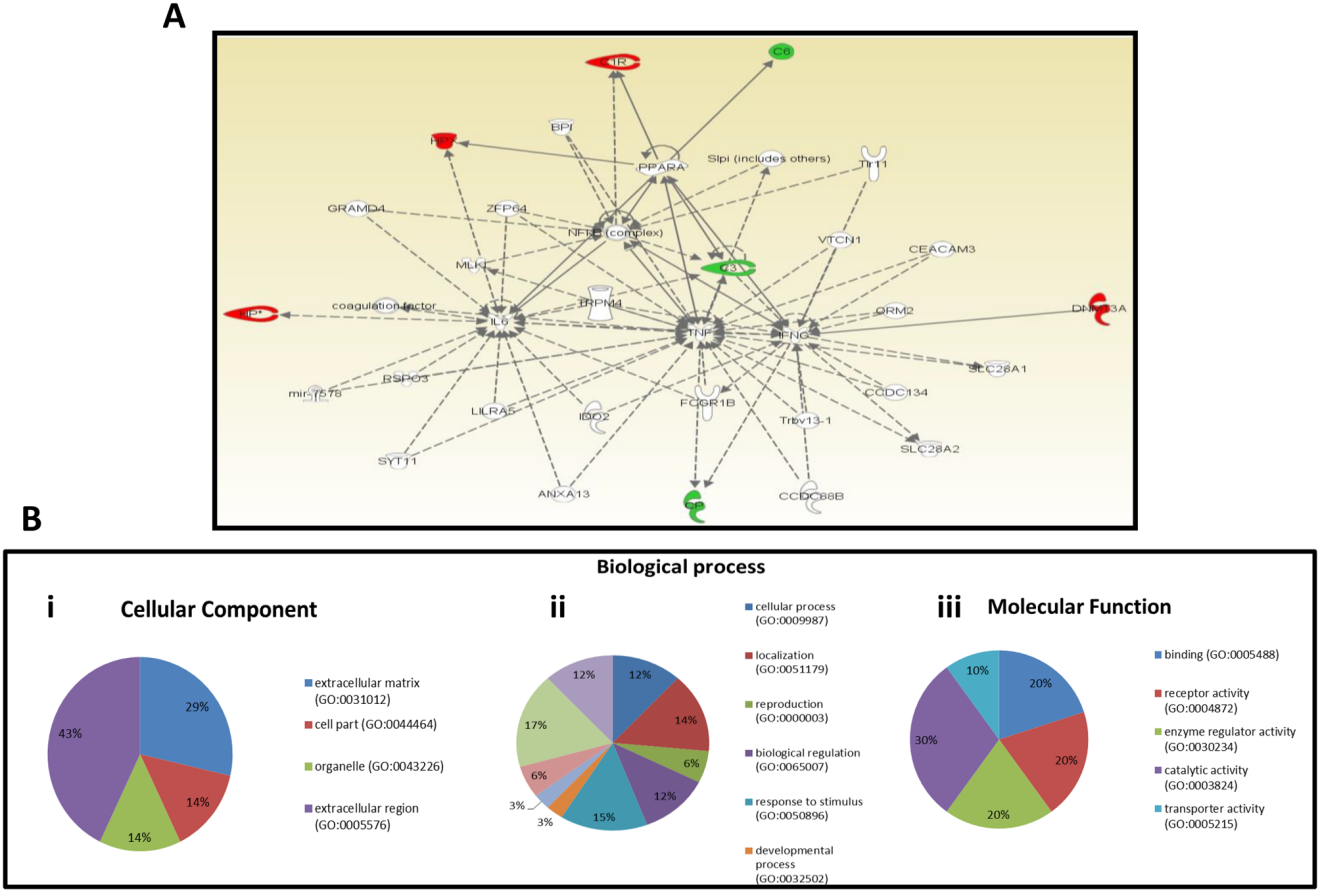
Protein interaction network and function. (**A**) Ingenuity Pathway Analysis (IPA) showing the major protein interaction network of identified proteins. Direct interactions are represented as solid lines, whereas indirect interactions appear as dotted lines. (**B**) A representative pie charts indicating the cellular localization (i), role of the identified proteins in the biological and physiological systems (ii) and the molecular functions of differentially identified proteins (iii). A total of six biological processes with their respective cellular locations were categorized.

We then sought to use the Ingenuity Pathways Analysis (IPA), which accurately predicts for interacting partner proteins and also determines the signal transduction in various pathways, to identify the biological and molecular protein network related to our proteins of interest. The IPA revealed the interaction of the differentially expressed proteins in different networks and biological processes associated mainly with immunological disease, inflammatory disease and inflammatory response with highest score 17 (Fig. 4A). In this network, the transcription factor (NF-kB) as well as the cytokines interleukin-6 (IL-6) and interferon gamma (IFN-γ), which are known to play a pivotal regulatory role in carcinogenesis, are connected to three of the enriched proteins. Functional annotation was carried out to identify the molecular functions of the differentially expressed proteins. The result revealed that 30% of the proteins are involved in catalytic activities, 20% are involved in transporter activities, 20% are involved in enzyme regulator activities, 20% in receptor activities and 10% in binding activities (Fig. 4B). In addition, the majority of these proteins are components of the cellular compartment, the organelles or the extracellular matrix compartment (Fig. 4B).

### Diagnostic performance of the plasma biomarkers

Among the three identified circulating serum protein biomarkers haptoglobin displayed the highest AUC > 0.87 (confidence interval 72–99%, *p* = 0.002) followed by C6 and ceruloplasmin with an AUC = 0.68 (confidence interval 46–89%, *p* = 0.39) and 0.68 (confidence interval 42–95%, *p* = 0.45) respectively (Supplementary Fig. 5). The combination of the haptoglobin, ceruloplasmin and C6 did not yield a better diagnostic efficacy for low grade cancer patients compared to individual biomarker alone (data not shown).

## Discussion

Bladder cancer (BC) is the second most common genitourinary neoplasm after prostate cancer, in men, with steady increase incidence rate^1^. This situation necessitates an urgent protocol to develop biomarkers for early detection and risk stratification.

The recent histopathological classification of bladder cancer cases by the World Health Organization (2016) into low grade and high grade has major implications on the risk stratification and the management of bladder cancer patients^22,23^. Interestingly, progression of low-grade NMIBC to MIBC is frequent (up to 20% of cases) and is critically important in the management of BC patients^24^. The high recurrence rates together with the disease heterogeneity and the limited treatment options for bladder cancer patients advocate the development of alternative approaches for early cancer detection.

A wide range of approaches have utilized for biomarker discovery including RNA-seq, micro‐RNA (miR), circulating tumor DNA (ctDNA), proteomics, metabolomics, and intact circulating tumor cells^25–29^. Interesting new biomarkers of disease activity have been identified however biomarker validation is difficult and only few have made a significant clinical impact to date^30^.

The body biological fluids, such as serum and plasma, represent ideal and important tools for the development of novel biomarker molecules distinguishing between health and disease.

In the current study we combined both 2D-DIGE and mass-spectrometry to investigate the proteomic plasma signature from patients with low grade NMIBC and healthy controls. Several plasma biomarkers were identified some of which were significantly up-regulated (Hemopexin, Haptoglobin, Kelch domain-containing protein 8B, E3 ubiquitin-protein ligase HECTD, DNA (cytosine-5)-methyltransferase 3A, Complement C1r subcomponent, Immediate early response 3-interacting protein 1, Integrator complex subunit 10, Complement component C6) other were significantly down-regulated (Ceruloplasmin, Complement C3) in plasma of patients with low grade bladder cancer compared to healthy controls (Table 2). Three of these proteins (Haptoglobin, Ceruloplasmin and Complement component C6) were selected for further validation in an independent cohort. Haptoglobin showed a significantly high expression in tumor samples compared to healthy controls (*p* = 0.0006) by western blotting. Three forms of haptoglobin have been identified which may indicate numerous potential splices variants and post-translational modification of haptoglobin. Our finding is consistent with previous ones reporting elevated haptoglobin levels in Turkish and Polish bladder cancer patients^17,31^. In a recent attempt to analyze the proteins content in the plasma samples of patients with bladder cancer and other inflammatory condition of the bladder, Lemańska et al. (2019) reported elevated levels of both haptoglobin and Complement C3 in cancer patients compared to healthy controls.

Under normal physiological conditions Haptoglobin stabilize hemoglobin (Hb) through binding and forming a complex with it. Haptoglobin has also significant protective properties against oxidative damage by Hb^32^. Is has been demonstrated that plasma haptoglobin level increased in several solid tumors including breast cancer, colon cancer, pancreatic cancer, lung cancer and ovarian cancer where it is considered as an independent prognostic marker^33–38^.

Our results indicated that the Complement component C6 protein expression was significantly down-regulated in patients with low grade tumors compared to control in the discovery set (*p* = 0.001) and the validation set (*p* = 0.046). To our knowledge this is the first study describing the association between elevated level of C6 and bladder cancer. Members of the complement system, consisting of over 50 plasma proteins, are potent effector of innate immunity involved in host defense^39^. Activation of Complement system leads to the generation of membrane attack complex. The latter requires the sequential assembly of complement proteins including C6, to form multi-protein pore involved in a direct lyses of a wide range of Gram-negative bacteria^40^. Recent experimental evidence indicated that activation of complement system plays an important role in carcinogenesis, however the exact mechanism is yet to be elucidated^41^.

As for ceruloplasmin, although the difference in protein expression level between tumors and controls did not reach significance, although there is a trend toward decrease protein expression in tumor compared to healthy controls (*p* = 0.29). Western blots of plasma samples probed with ceruloplasmin antibody showed expected 140 kDa isoform in healthy controls. Interestingly, cancer patients express another 80 kDa band that is differentially expressed across cancer patients. Patients expressing no or low levels of 80 kDa band show strong expression of the 140 kDa band whereas patients expressing high levels of 80 kDa band exhibit no expression of the 140 kDa band. The low molecular weight isoform is completely absent in healthy controls. This suggests that the 80 kDa isoform of the ceruloplasmin protein represent a posttranslational cleaved product or produced in a pre-spliced transcript. It may also arise from un-characterized ceruloplasmin isoforms. Ceruloplasmin is a secreted glycoprotein member of the multi-copper oxidase family involved in a wide variety of cellular processes mainly in copper transport and ion metabolism^42^. It also catalyzes the oxidation of polyamines, catecholamines and polyphenols. Ceruloplasmin loss-of-function is involved in neurodegenerative syndrome, whereas high ceruloplasmin level is associated with copper toxicity, Alzheimer’s disease and cardiovascular diseases^43,44^. As ceruloplasmin levels were significantly high in cancer, ceruloplasmin has been suggested to be potential biomarkers in numerous solid tumors^45–50^. The relationship between ceruloplasmin level and bladder cancer has not been documented previously. In a meta-analysis by Mao and Huang, the authors reported a marked increase in serum copper level in patients with bladder cancer compared with controls which might be caused by excess of Cu-binding components, such as ceruloplasmin^51^. Taken together, our findings support the possible involvement of high plasma ceruloplasmin level in increasing copper levels in the blood in bladder cancer patients. The exact mechanism of ceruloplasmin action in bladder carcinogenesis is yet to be elucidated. Furthermore, network analysis using IPA identified cancer-associated network associated with the differentially expressed proteins. The data presented show that three of seven proteins (ceruloplasmin, Complement C3 and Complement C6) are functionally implicated in immune disease and inflammatory response, DNA replication and cellular assembly and organization. Deregulation of these proteins has also been implicated in cancer development and progression.

## Conclusion

We believe that differential plasma proteome analysis of patients with low grade BC provides new insights on disease pathophysiology. The current study sheds the light on new plasma marker proteins that could potentially help in early detection of bladder cancer. The level of haptoglobin was found to be highly elevated in low grade NMIBC patients, which point to the potential involvement of this protein in early stages of bladder cancer development. Its utility as a non-invasive biomarker tool in the context of bladder cancer warrant further in‐depth investigation. There are several limitations to this study. Although the analysis was performed using an independent validation cohort with a freshly collected plasma samples, the results should be considered as preliminary given that the small sample size hence the results merit validation in a larger multicenter study. Also, the clinicopathological features such as smoking status, treatment and survival were not available at the time of the analysis which prevents us from conducting more relevant correlations and consistent comparison.

## Methods

### Patient samples

Blood samples were collected from patients with low grade bladder cancer attending King Abdulaziz University Hospital (KAUH) and King Faisal specialist hospital and Research Center (KFSH&RC), Jeddah, Saudi Arabia. This study was conducted in accordance with the Declaration of Helsinki and was approved by the Unit of biomedical Ethics, Research Ethics Committee, King Abdulaziz University Hospital (reference No. 149-04) and by the institutional Review Board Committee, King Faisal specialist hospital and Research Center, Jeddah (reference No: RC-J/36/36). Written informed consent was obtained from all the participants.

### Plasma samples collection and depletion

Blood samples were collected *in EDTA-*containing tubes and centrifuged at 1,500 × *g* for 10 min at 4 °C. Plasma was carefully removed and aliquots were stored at − 80 °C until use.

For proteomic analysis, Top-20 Depletion ProteoPrep column (Sigma, USA) was used to deplete high abundance proteins in the collected plasma samples as per the manufacturer’s instructions and protocol.

### 2D DIGE labeling

Depleted plasma samples were subjected to TCA/acetone precipitation. Protein labeling was performed as described previously^52^. Briefly, depleted plasma samples were mixed (1:4 ratio) with ice-cold acetone containing 10% w/v TCA and vortexed for 15 s for uniform mixing. The mixture was incubated over night at − 20 °C for protein precipitation. After incubation, tubes were centrifuged at 2000*g* for 15 min at 4 °C. Supernatants were discarded and the pellet solubilized in labeling buffer (7 M urea, 2 M thiourea, 30 mM Tris–HCl, 4% CHAPS, pH 8.5). The proteins were then labeled with 400 pmol of CyDye DIGE Fluor dyes. Each technical duplicate of the sample was covalently labeled with fluorophores Cy3, Cy5 or Cy2 (internal standard). First dimension analytical gel electrophoresis was performed as follows. Rehydration followed by isoelectric focusing. The strips were then equilibrated and separated on 12.5% (SDS-PAGE) gels using an Ettan Dalt Six device. The gels were scanned with a Typhoon 9400 scanner using appropriate wavelengths and filters for Cy2, Cy3 and Cy5. Difference in-gel electrophoresis (DIGE) images were analyzed using the Progenesis SameSpots v.3.3 software (Nonlinear Dynamics Ltd., UK) as described previously^53^. All spots were pre-filtered and manually checked before applying the statistical criteria (ANOVA test, p ≤ 0.05 and fold ≥ 1.5). Normalized spot volumes, instead of spot intensities, were used in statistical processing. Only those spots that fulfilled the abovementioned statistical criteria were submitted for MS analysis.

### Protein identification by MALDI-TOF mass spectrometry (MS)

Coomassie stained gel spots were excised manually, washed and digested overnight, at 37 °C according to the previously described protocol^52^. The digests were transferred to a 0.5 mL tube and the peptides were extracted by the addition of 30% CH_3_CN containing 0.1% CF_3_COOH^54^. An aliquot of the digestion solution (1 μL) was spotted onto a MALDI target together with 0.5 μL of a matrix CHCA in 1 μL of 30% CH_3_CN and 0.1% aqueous CF_3_COOH and then left to dry before MS analysis. Protein identification was assigned by peptide mass fingerprinting (PMFs) using an ultrafleXtreme time-of-flight mass spectrometer and confirmed by MS/MS analysis of at least three peptides in each sample using LIFT spectra performed by MALDI-TOF–MS/MS. The MS data were interpreted by using BioTools v.3.2, together with the Mascot search algorithm. Identified proteins were accepted as correct if they showed a Mascot score greater than 56 and p < 0.05.

### Pathway analysis

The quantitative data were then imported into IPA software (Ingenuity Systems, https://www.ingenuity.com). This software aids in determining the functions and pathways most strongly associated with the protein list by overlaying experimental expression data on networks constructed from published interactions.

### Western blot analysis

Protein samples were separated on 8% SDS-polyacrylamide gels. Proteins were transferred to nitrocellulose membranes and blocked with 5% non-fat milk then incubated with the primary antibodies overnight at 4 °C. Membranes were then incubated with HRP-labeled secondary antibodies and bands were visualized with enhanced chemiluminescence reagent. To ensure equal gel loading, equal amounts of plasma samples were separated using 8% PAGE gels and stained with Coomassie blue stain.

### Statistical analysis

Statistical analyses were performed using Fisher’s exact test. A *p*-value less than 0.05 was considered statistically significant. Data analysis was performed using SPSS (SPSS, V.25, USA).

### Ethical approval

This study was ethically approved by the Research Ethics Committee at KAUH (reference No. 149-04) and by the institutional Review Board Committee at KFSH&RC (reference No: RC-J/36/36).

## Supplementary information

Supplementary figure 1

Supplementary figure 2

Supplementary figure 3

Supplementary figure 4

Supplementary figure 5

Supplementary table 1

Supplementary information

## Data Availability

The datasets generated during and/or analyzed during the current study are available from the corresponding author on reasonable request.
