# Supplementary material for: Circulating proteomic signature for detection of biomarkers in bladder cancer patients
Source: Sci Rep. 2020 Jul 3;10:10999. doi: 10.1038/s41598-020-67929-z (PMC7335182; doi:10.1038/s41598-020-67929-z)
Supplement: Supplementary file 1 — Supplementary figure 1 [file 41598_2020_67929_MOESM1_ESM.pptx]

## Slide 1
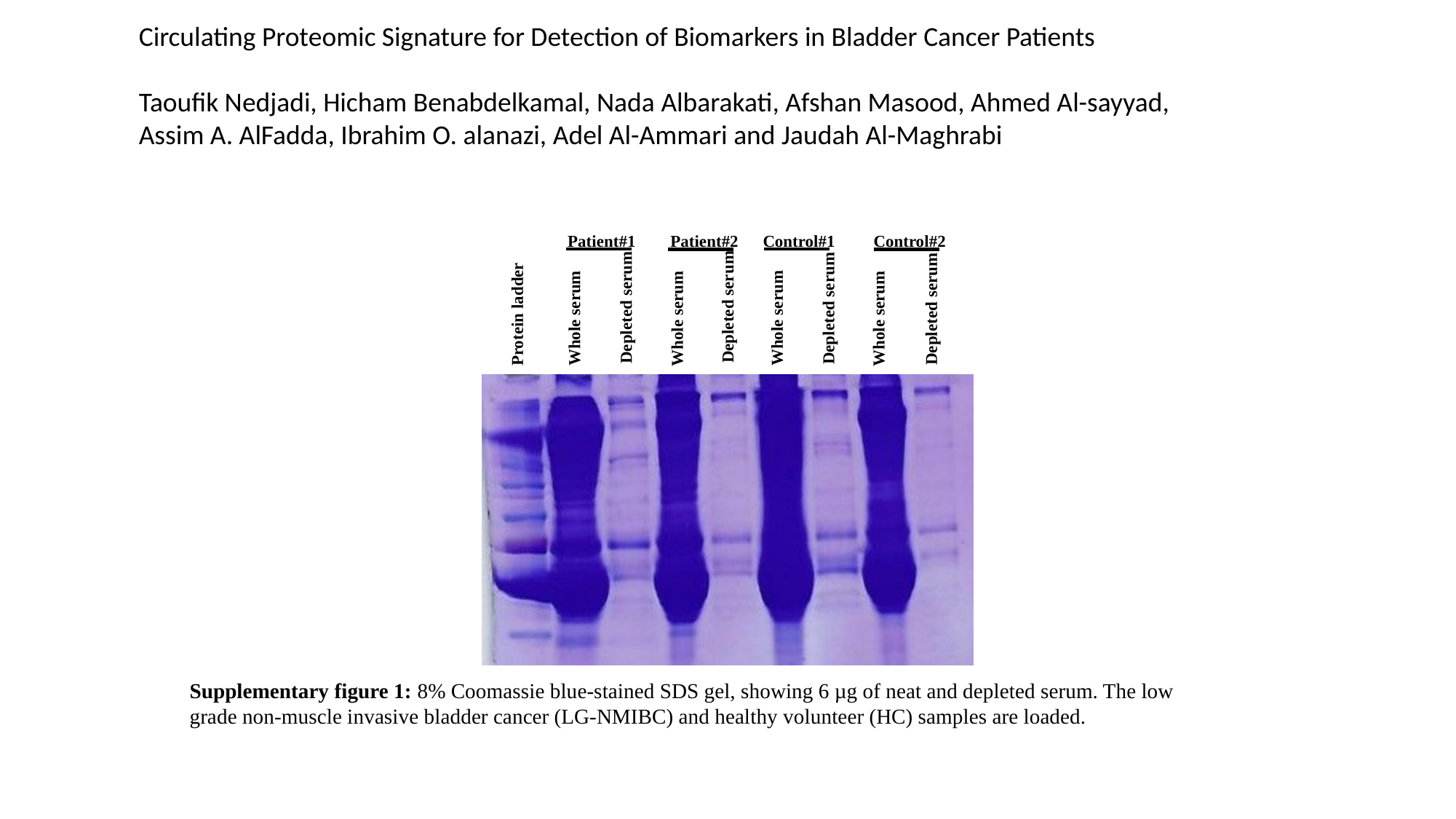

Circulating Proteomic Signature for Detection of Biomarkers in Bladder Cancer Patients
Taoufik Nedjadi, Hicham Benabdelkamal, Nada Albarakati, Afshan Masood, Ahmed Al-sayyad, Assim A. AlFadda, Ibrahim O. alanazi, Adel Al-Ammari and Jaudah Al-Maghrabi
Patient#1
Patient#2
Control#1
Control#2
Protein ladder
Depleted serum
Depleted serum
Depleted serum
Depleted serum
Whole serum
Whole serum
Whole serum
Whole serum
Supplementary figure 1: 8% Coomassie blue-stained SDS gel, showing 6 µg of neat and depleted serum. The low grade non-muscle invasive bladder cancer (LG-NMIBC) and healthy volunteer (HC) samples are loaded.
