# Supplementary material for: Circulating proteomic signature for detection of biomarkers in bladder cancer patients
Source: Sci Rep. 2020 Jul 3;10:10999. doi: 10.1038/s41598-020-67929-z (PMC7335182; doi:10.1038/s41598-020-67929-z)

## Slide 1
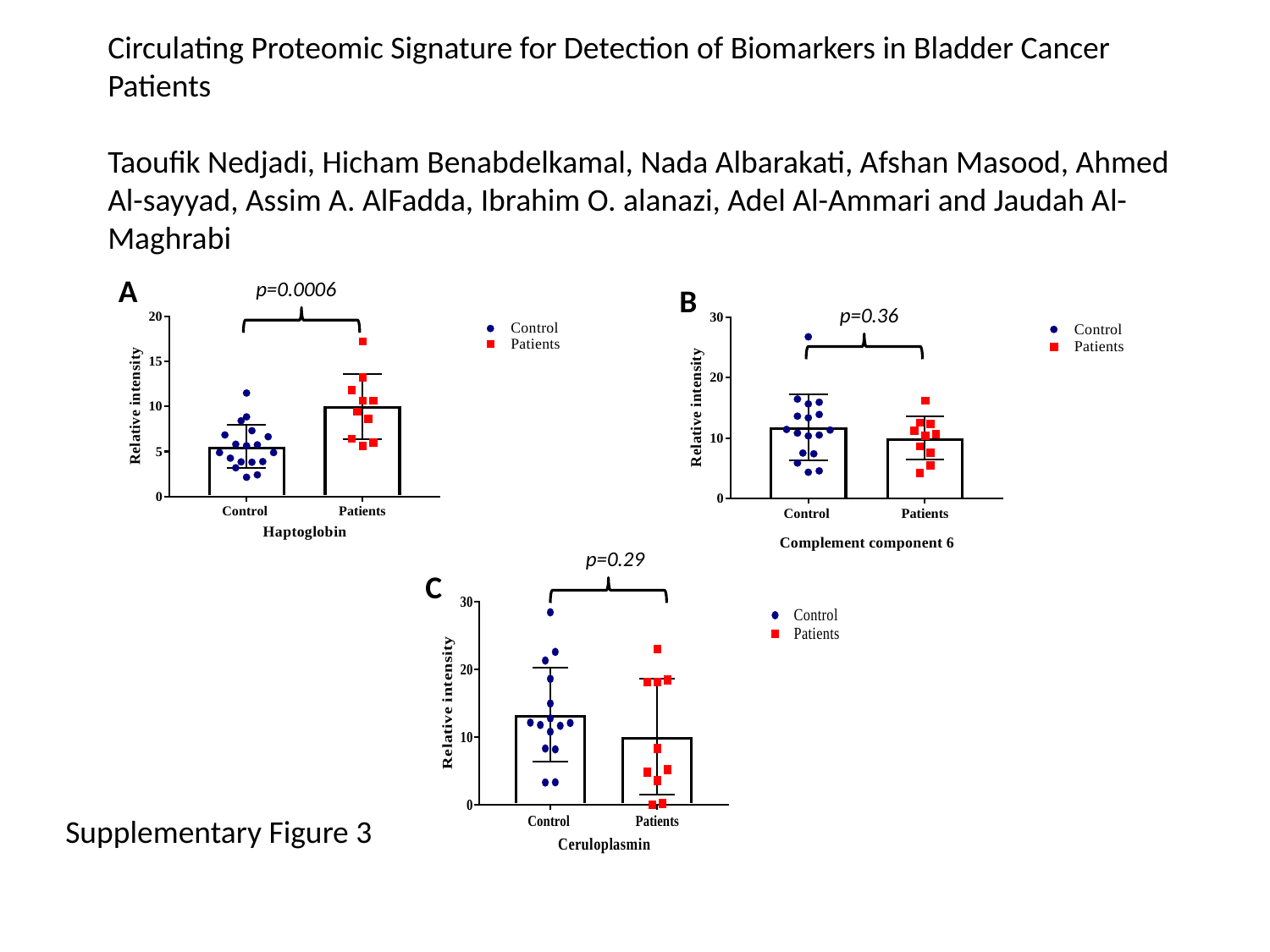

Circulating Proteomic Signature for Detection of Biomarkers in Bladder Cancer Patients
Taoufik Nedjadi, Hicham Benabdelkamal, Nada Albarakati, Afshan Masood, Ahmed Al-sayyad, Assim A. AlFadda, Ibrahim O. alanazi, Adel Al-Ammari and Jaudah Al-Maghrabi
A
p=0.0006
B
p=0.36
p=0.29
C
Supplementary Figure 3

## Slide 2
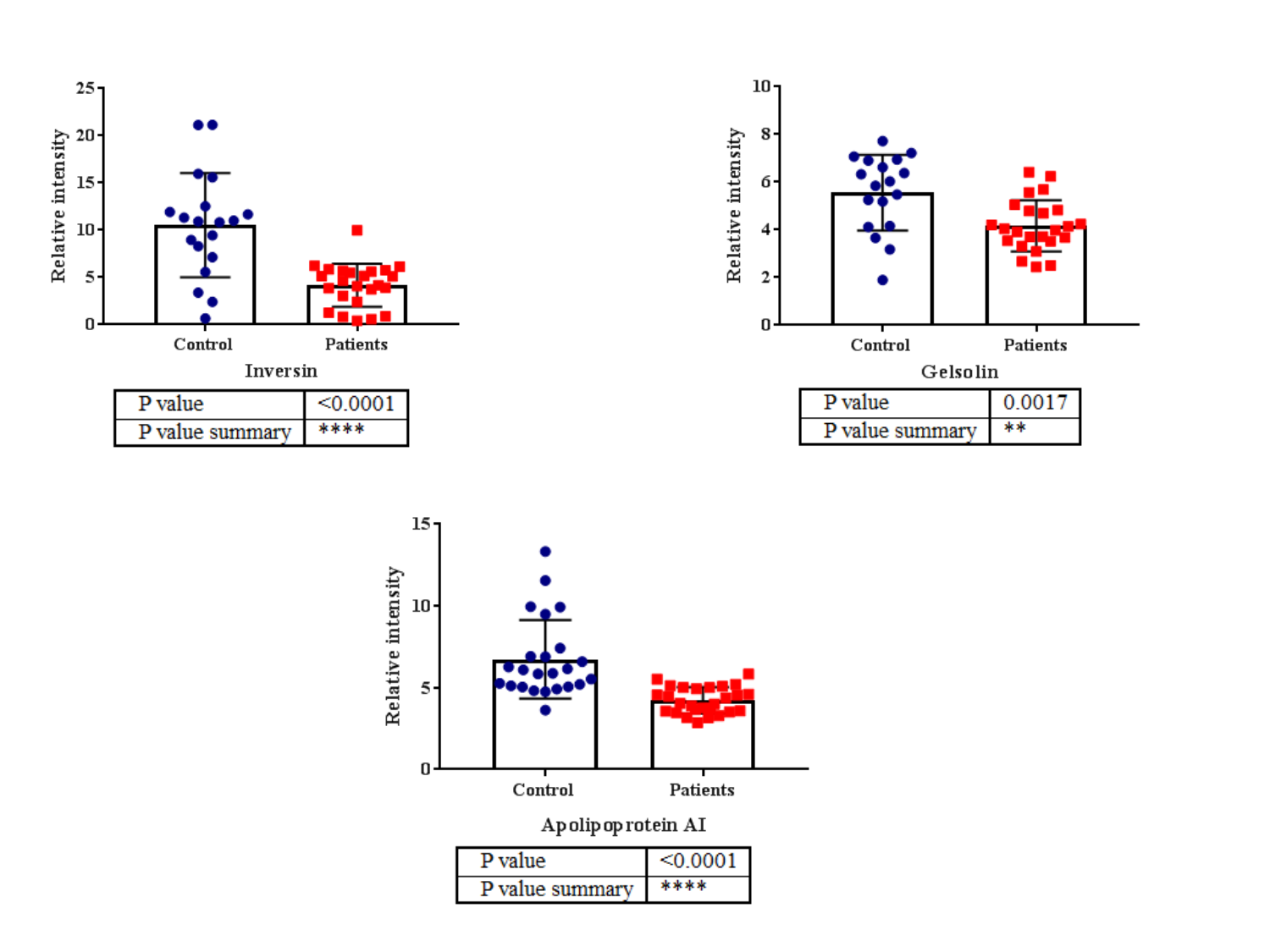

Supplement: Supplementary file 3 — Supplementary figure 3 [file 41598_2020_67929_MOESM3_ESM.pptx]
