# Supplementary material for: Circulating proteomic signature for detection of biomarkers in bladder cancer patients
Source: Sci Rep. 2020 Jul 3;10:10999. doi: 10.1038/s41598-020-67929-z (PMC7335182; doi:10.1038/s41598-020-67929-z)
Supplement: Supplementary file 6 — Supplementary table 1 [file 41598_2020_67929_MOESM6_ESM.docx]

| Variables | Discovery set | Validation set | Significance |
| --- | --- | --- | --- |
| **Age**  Mean (range) | 59.75 (55-69) | 60.5 (50-73) | ns |
| **Gender**   - Male - Female | 3  1 | 8  2 | ns |
| **Marital status**   - Married - Not married | X | X | ns |
| **Grade** | Low grade | Low grade | ns |
| **Stage**   - pT0 - pT1 | 1  3 | 2  8 | ns |
| **Tumour type**   - transitional - squamous | X | X | ns |
| **Tumour sub-type**   - papillary - flat | X | X | ns |
| **Vascular metastasis**   - yes - no | X | X | ns |
| **Lymph node met**   - yes - no | X | X | ns |
| **Recurrence**   - yes - no | X | X | ns |
| **Family history**   - yes - no | X | X | ns |
| **Smoking**   - Yes - No | 3  1 | 7  3 | ns |
| **Survival**   - Alive - Deceased | X | X | ns |

Circulating Proteomic Signature for Detection of Biomarkers in Bladder Cancer Patients

Taoufik Nedjadi, Hicham Benabdelkamal, Nada Albarakati, Afshan Masood, Ahmed Al-sayyad, Assim A. AlFadda, Ibrahim O. alanazi, Adel Al-Ammari and Jaudah Al-Maghrabi
