# Supplementary material for: Circulating proteomic signature for detection of biomarkers in bladder cancer patients
Source: Sci Rep. 2020 Jul 3;10:10999. doi: 10.1038/s41598-020-67929-z (PMC7335182; doi:10.1038/s41598-020-67929-z)
Supplement: Supplementary file 7 — Supplementary information [file 41598_2020_67929_MOESM7_ESM.pptx]

## Slide 1
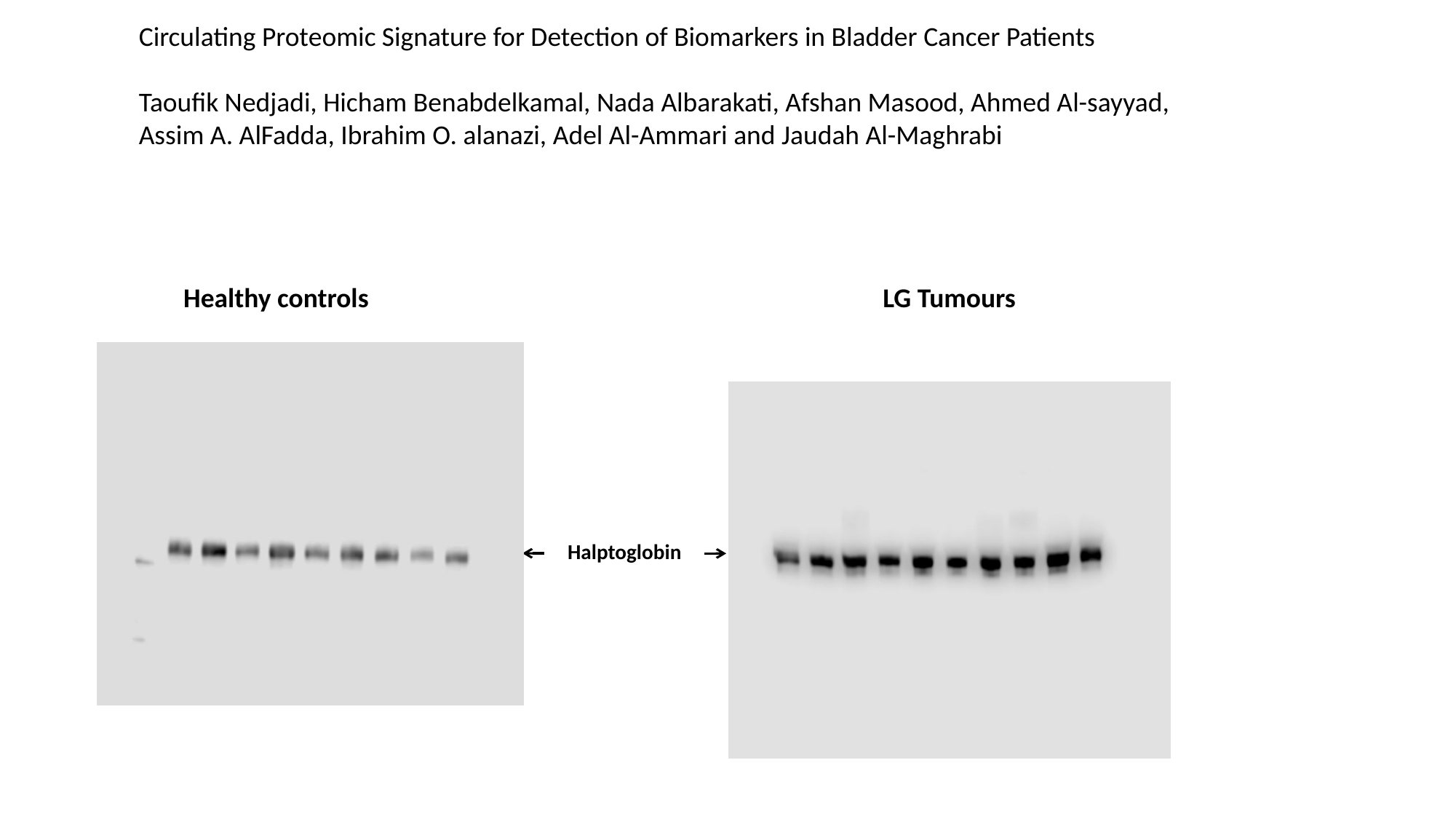

Circulating Proteomic Signature for Detection of Biomarkers in Bladder Cancer Patients
Taoufik Nedjadi, Hicham Benabdelkamal, Nada Albarakati, Afshan Masood, Ahmed Al-sayyad, Assim A. AlFadda, Ibrahim O. alanazi, Adel Al-Ammari and Jaudah Al-Maghrabi
Healthy controls
LG Tumours
Halptoglobin

## Slide 2
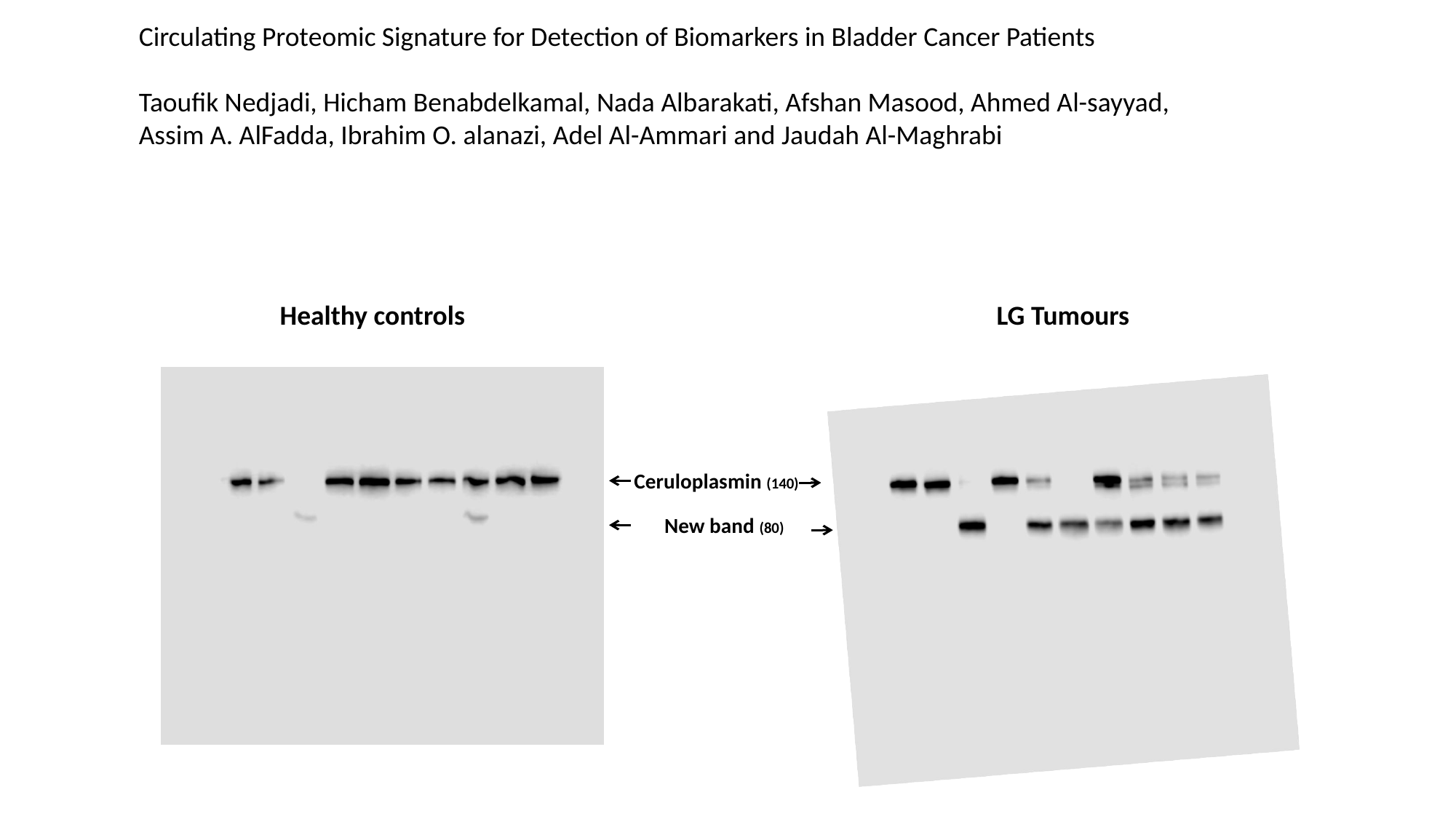

Circulating Proteomic Signature for Detection of Biomarkers in Bladder Cancer Patients
Taoufik Nedjadi, Hicham Benabdelkamal, Nada Albarakati, Afshan Masood, Ahmed Al-sayyad, Assim A. AlFadda, Ibrahim O. alanazi, Adel Al-Ammari and Jaudah Al-Maghrabi
Healthy controls
LG Tumours
Ceruloplasmin (140)
New band (80)

## Slide 3
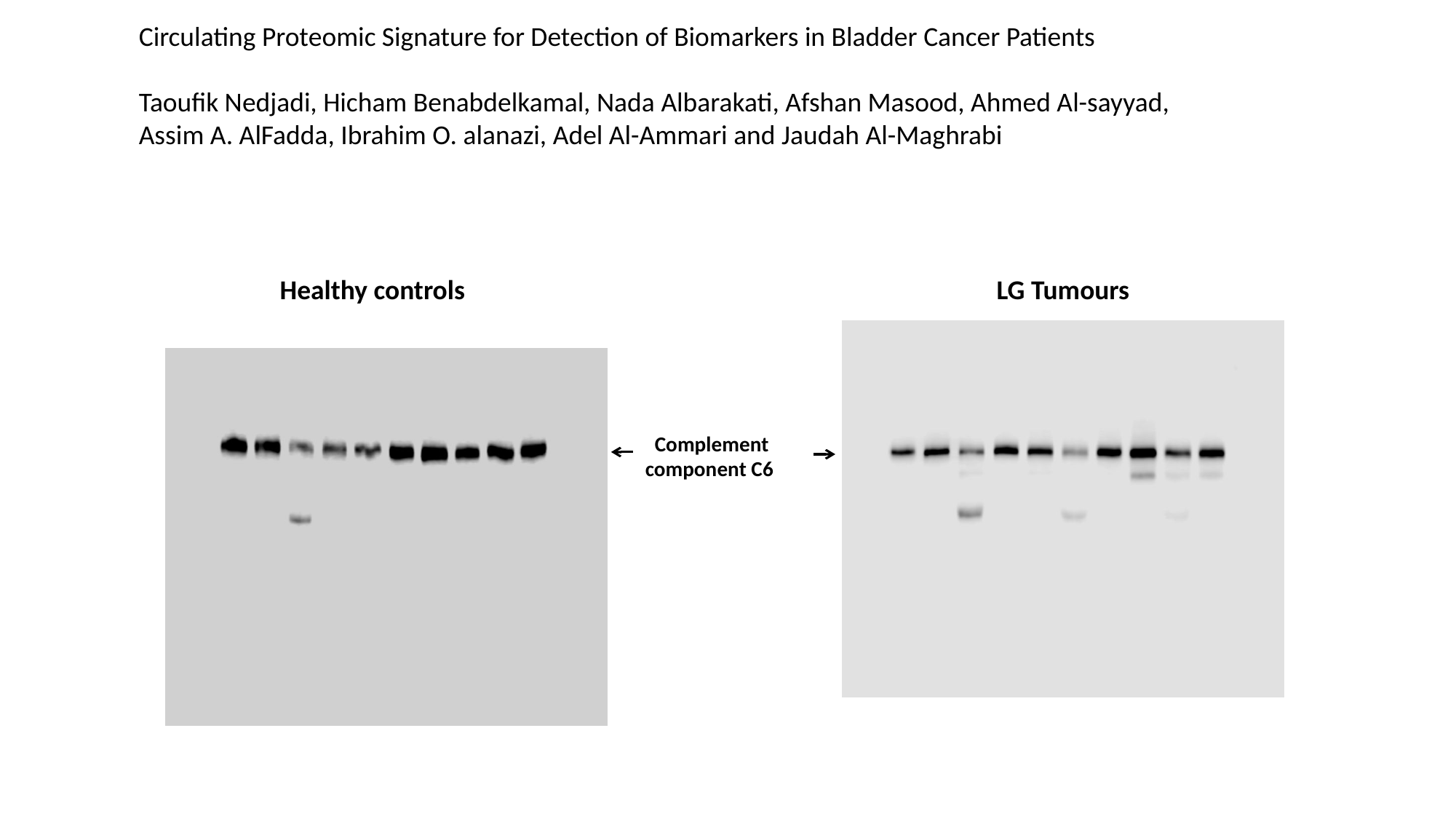

Circulating Proteomic Signature for Detection of Biomarkers in Bladder Cancer Patients
Taoufik Nedjadi, Hicham Benabdelkamal, Nada Albarakati, Afshan Masood, Ahmed Al-sayyad, Assim A. AlFadda, Ibrahim O. alanazi, Adel Al-Ammari and Jaudah Al-Maghrabi
Healthy controls
LG Tumours
Complement component C6
